# Large Renal Abscess in Pregnancy: Case Report of a Rare Finding

**DOI:** 10.7759/cureus.35610

**Published:** 2023-02-28

**Authors:** Ikechukwu E Eze, Aisha R Ahmed, Claudia Gyimah, Oluwatobi G Lasisi, Uzoamaka Nwaogwugwu

**Affiliations:** 1 Internal Medicine, Howard University Hospital, Washington DC, USA; 2 Obstetrics and Gynecology, Howard University Hospital, Washington DC, USA; 3 Family Medicine, Howard University Hospital, Washington DC, USA; 4 Nephrology, Howard University Hospital, Washington DC, USA

**Keywords:** urinary tract infection, kidney abscess, pregnancy, high-risk pregnancy, acute pyelonephritis, renal abscess

## Abstract

The incidence of renal abscesses during pregnancy has not been well-established. A renal abscess is usually secondary to the complications of acute pyelonephritis and can lead to severe consequences, including fetal and/or maternal death. Little is known about the incidence of renal abscesses in pregnant women; however, the literature consistently refers to it as an extremely rare occurrence. We report a case of a large renal abscess discovered in the early postpartum period following a recurrent urinary tract infection and flank pain during pregnancy. The patient was successfully managed with abscess drainage and prolonged antibiotics.

## Introduction

The incidence of renal abscesses during pregnancy is not well-established. However, this is a rare condition. When it occurs, it is usually secondary to the complications of acute pyelonephritis and can lead to severe consequences, including fetal and/or maternal death [1,2]. Here, we report a case of a large renal abscess discovered in the early postpartum period following recurrent urinary tract infection and flank pain during pregnancy.

## Case presentation

We present the case of a 36-year-old woman who had had three previous pregnancies and one full-term delivery. She was admitted to our hospital at a gestational age of 37 weeks for uncontrolled hypertension. Her antepartum history was significant for gestational hypertension and recurrent urinary tract infection, with positive Escherichia coli on a urine culture. She was initially treated with ampicillin and was on suppressive therapy with nitrofurantoin at the time of her presentation for labor and delivery. She subsequently developed preeclampsia with severe features (systolic blood pressure > 180s) and was febrile (temperature, 100.6F) with concurrent fetal tachycardia. She delivered a viable male fetus via cesarean section.

On postpartum day 0, she was also noted to have anemia, with postoperative hemoglobin and hematocrit of 6.9 and 21.7, respectively. Her white blood cell (WBC) count was elevated (12.00 - 15.09 X 10E9). She required a total of three units of packed red blood cell (RBC) transfusion postpartum. On postpartum day 1, the patient was noted to have a fever (100.9F), right fundal tenderness, and right costovertebral angle tenderness. Endometritis was suspected and antibiotic therapy was initiated with ampicillin, gentamicin, and clindamycin.

**Figure 1:**
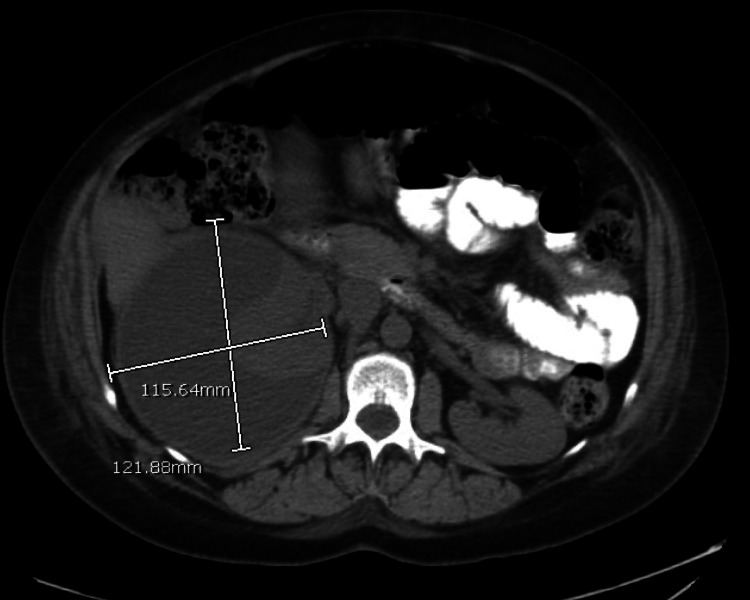
Axial CT showing dimensions of the renal abscess

**Figure 2:**
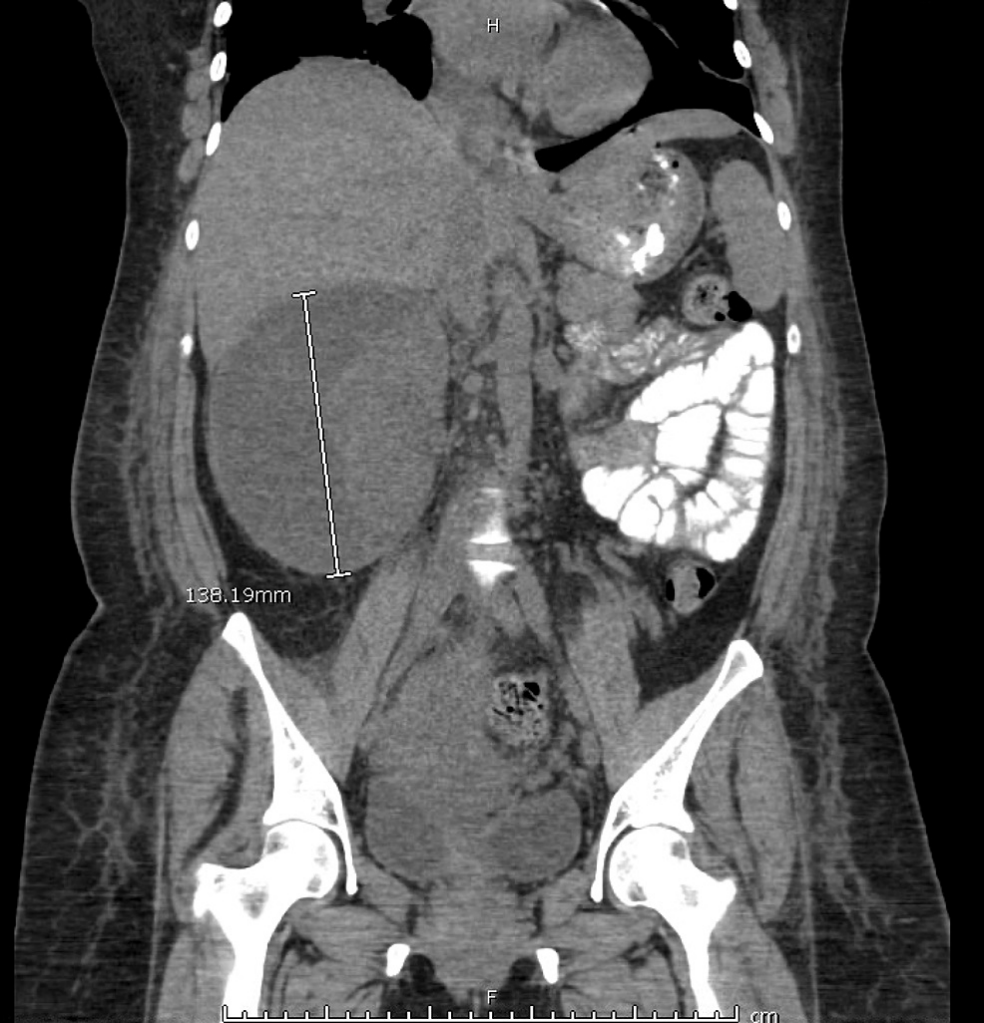
Coronal CT image of the renal abscess

She continued to be febrile despite antibiotic treatment (100.9 - 102.7F) and was also found to have hematuria on urinalysis. Renal ultrasonography (US) on postpartum day 2 revealed a large complex right perinephric fluid collection that could represent a resolving hematoma, urinoma, or inflammatory collection. Computed tomography (CT) of the abdomen and pelvis with contrast done on the same day revealed a cystic structure on the right kidney measuring 13.8 x 12.2 x 11.6 cm reported as a possible urinoma versus abscess (Figures 1, 2). Antibiotics were changed to cefepime at this point, given worsening leukocytosis and continued fever.

Interventional radiology (IR)-guided aspiration and drainage of the right renal abscess were performed on postpartum day 3. A 10 French drainage was placed with an initial effluent of 730 ml of cloudy serous fluid. Fluid analysis was significant for 46,840 WBCs and 5000 RBCs. Fluid culture showed gram-negative rods. Following abscess drainage, the patient was placed on ceftriaxone under the guidance of infectious disease specialists. The abscess culture grew E. coli resistant to ampicillin, ciprofloxacin, and sulfamethoxazole/trimethoprim. She underwent cystoscopy, fluoroscopy, and retrograde pyelography to rule out a urinary tract injury on postpartum day 4. She was switched from intravenous cefepime to ceftriaxone, which was continued for 14 days after the initial drainage of the abscess.

A subsequent renal ultrasound showed markedly reduced collection five days after drain placement. Her drain was removed as an outpatient following a satisfactory sonogram. The patient was hemodynamically stable and discharged with an appropriate follow-up schedule. During the one-month and six-month follow-ups, she was found to have made a full clinical, biochemical, and radiological recovery.

## Discussion

The index patient developed a right renal abscess following a urinary tract infection and pyelonephritis. This is the known natural history of renal abscess development. Acute pyelonephritis has been reported in up to 2% of pregnancies [3]. Previous infection is a known risk factor for recurrence [4]. Little is known about the incidence of renal abscesses in pregnant women, but it is consistently documented in the literature as a rare occurrence. A systematic review of acute pyelonephritis by Grette et al. revealed that renal abscesses occur in 6% of pregnant women with acute pyelonephritis [1]. In pregnancy, structural and functional changes are thought to contribute to higher rates of urinary tract infection and pyelonephritis. However, this does not translate into a higher incidence of renal abscesses. This rarity may be due to guideline-based treatment of asymptomatic bacteriuria in pregnancy, as well as structured antenatal care, giving pregnant women access to care and opportunities for the management of known predisposing conditions such as urinary tract infections and acute pyelonephritis.

Similar to the index case, renal abscesses occurred more commonly on the right side, likely because of dextrorotation of the uterus and urinary stasis caused by ureteral compression by the gravid uterus. E coli remains the most frequent cause of acute pyelonephritis and is most likely isolated from abscess cultures, as seen in this case [5].

Ultrasound imaging is the most common modality and test of choice during pregnancy. Abdominal CT with contrast may better characterize abscesses and can be used in non-pregnant or post-partum women. Serial USG and CT scans of the abdomen and pelvis were used in our case. MRI remains extensively used in obstetrics because of its safety profile for both the mother and fetus while simultaneously avoiding radiation and providing good contrast imagery for diagnosing renal and perirenal abscesses [6].

Treatment is dependent on the size of the abscess; abscesses ≤ 3 cm are considered small and can be managed conservatively with antibiotics, whereas those ≥ 5 cm are generally managed with percutaneous drainage in addition to intravenous antibiotic therapy [7]. The index patient had a large abscess, which was subsequently managed with percutaneous drainage. The recommended antibiotic is a third-generation cephalosporin prior to sensitivity due to the prevalence of E. Coli as the causative organism. In this case, the patient was administered ceftriaxone 2 g IV for at least two weeks after percutaneous drainage and drain removal. Although the index case achieved full recovery without significant maternal or fetal complications, cases of fatal outcomes have been described in the literature [2].

## Conclusions

Renal abscesses, although rare, can cause serious complications in pregnancy. It requires prompt diagnosis and treatment. Appropriate treatment of UTIs (and asymptomatic bacteriuria) in pregnancy is the mainstay of prevention. Considering the rarity of renal abscesses in pregnancy as well as the possibility of fatal outcomes, it is imperative to have a high index of suspicion in patients with a suggestive history of urinary tract infection and/or pyelonephritis.
